# Autotetraploidy of rice does not potentiate the tolerance to drought stress in the seedling stage

**DOI:** 10.1186/s12284-024-00716-w

**Published:** 2024-06-18

**Authors:** Shunwu Yu, Tianfei Li, Xiaoying Teng, Fangwen Yang, Xiaosong Ma, Jing Han, Li Zhou, Zhijuan Bian, Haibin Wei, Hui Deng, Yongsheng Zhu, Xinqiao Yu

**Affiliations:** 1 Shanghai Agrobiological Gene Center, Shanghai, 201106 China; 2 Key Laboratory of Grain Crop Genetic Resources Evaluation and Utilization, Ministry of Agriculture and Rural Affairs, Shanghai, 201106 China; 3 Institute of Crop Sciences, Wuhan Acadamy of Agricultual Sciences, Wuhan, 430345 China

## Abstract

Polyploid is considered an advantage that has evolved to be more environmentally adaptable than its diploid. To understand if doubled chromosome of diploid rice can improve drought tolerance, we evaluated the diploid (2X) and autotetraploid (4X) plants of three *indica* and three *japonica* varieties. Drought stress in the plastic bucket of four-leaf stage revealed that the drought tolerance of 4X plants was lower than that of its diploid donor plants. The assay of photosynthetic rate of all varieties showed that all 4X varieties had lower rates than their diploid donors. The capacity for reactive oxygen species production and scavenging varied among different 2X and 4X varieties. Further, transcriptomic analysis of 2X and 4X plants of four varieties under normal and drought condition showed that the wide variation of gene expression was caused by difference of varieties, not by chromosome ploidy. However, weighted gene co-expression network analysis (WGCNA) revealed that the severe interference of photosynthesis-related genes in tetraploid plants under drought stress is the primary reason for the decrease of drought tolerance in autotetraploid lines. Consistently, new transcripts analysis in autotetraploid revealed that the gene transcription related with mitochondrion and plastid of cell component was influenced most significantly. The results indicated that chromosome doubling of diploid rice weakened their drought tolerance, primarily due to disorder of photosynthesis-related genes in tetraploid plants under drought stress. Maintain tetraploid drought tolerance through chromosome doubling breeding in rice needs to start with the selection of parental varieties and more efforts.

**Supplementary Information:**

The online version contains supplementary material available at 10.1186/s12284-024-00716-w.

## Background

In nature, plants cannot move, and must endure adverse abiotic environmental conditions such as drought, salinity, extreme temperatures and nutrient deficiencies. Polyploidy or whole genome duplication is a ubiquitous phenomenon in plant species evolution facing environmental challenge (Wendel 2000; Paterson 2005; Soltis and Soltis 2009; Van de Peer et al. 2017, 2021). With genome doubling, multiple sets of genetic materials increase adaptability to environmental variability, and polyploidy plays a predominant role in bursts of adaptive speciation (Alix et al. 2017). Two main types of recent polyploidy can be distinguished. Autopolyploidy defines duplication of one genome within one species, which results in homologous chromosome sets in the cell (such as AA doubles to become AAAA). Allopolyploidy defines duplication of two or more divergent genomes in a single nucleus following interspecific hybridization, resulting in homologous chromosome sets in the cell (such as AA and BB become AABB) (Soltis and Soltis 2009; Alix et al. 2017). Numerous studies have revealed that polyploid species may have different ecological tolerances from their diploid progenitors (Ramsey 2011; Stuessy and Weiss-Schneeweiss 2019).

In evolution, polyploid has more tolerance to stress than their diploid, suggesting to be associated with increased adaptation to abiotic stress(Wendel 2000). In an outdoor drought and heat-stressed experiment about the perennial grass *Themeda triandra* Forssk., the yield of tetraploid plants was over four times higher than of diploids, despite being equal under more favorable growing conditions (Godfree et al. 2017). In the study of citrus, some tetraploid genotypes, appeared resistant compared to the other genotypes by the lesser decrease in photosynthetic capacity and the lower accumulation of oxidative species in roots and leaves, especially after long-term nutrient deficiency (Oustric et al. 2019). Moreover, people compared diploid species with tetraploid lines through directly doubling the number of chromosomes. Tetraploid plants obtained by colchicine treatment of shoots propagated in vitro possess a stronger antioxidant defense system and an increased heat tolerance compared with the diploid plants (Zhang et al. 2010). Tetraploidy of *Arabidopsis thaliana* decreases transpiration rate and potentiates plant tolerance to salt (Del and Ramirez-Parra 2014).

Rice is a staple food for more than half of the world’s population. One of many strategies for increasing the yield potential is rice autopolyploidy, especially autotetraploid hybrids. Autotetraploid hybrids showed a higher and positive heterobeltiosis and competitive heterosis than diploid hybrids, especially for grain yield (Wu et al. 2013). Abiotic tolerance is another desired characteristic. Autotetraploid rice from diploid *indica* rice 9311 exhibited a stronger tolerance to saline and alkaline stress than its diploid precursor (Wang et al. 2022a, b; Zhang et al. 2022). Autotetraploid rice from *japonica* rice Nipponbare, HN2026 and 02428 is also more tolerant to salt stress than its diploid precursor (Jiang et al. 2013; Wang et al. 2021). In these studies, the analysis of biochemical and physiological properties and differentially expressed genes (DEGs) indicated that autotetraploid under salt stress possibly possessed higher osmotic regulation ability, and higher antioxidant capacity, as well as lignin biosynthesis (Wang et al. 2022a; Zhang et al. 2022). Ploidy-induced genomic changes are strongly associated with epigenetic gene regulation. Tetraploidy induced DNA hypomethylation and potentiated JA-related genes for higher induction levels in rapid and robust responses to salt stress. After stress, elevated expression of stress-responsive genes in tetraploid rice can induce hypermethylation and suppression of transposable elements (TEs) adjacent to stress-responsive genes (Wang et al. 2021). However, a few reports have demonstrated drought tolerance of autotetraploid rice.

Can tetraploid rice also improve the drought tolerance compared to diploid rice? The previous study in 2014 indicated that the chromosome-doubling enhanced rice photoinhibition tolerance under drought stress and reduced MDA (malondialdehyde) content and superoxide anion production rate in two autotetraploid rice lines compared with the corresponding diploid rice lines (Yang et al. 2014). However, the study did not describe if the two autotetraploid rice lines possessed greater tolerance to drought than the corresponding diploid rice lines. In this study, three *japonica* and three *indica* rice varieties were used to evaluate drought tolerance of diploid rice and autotetraploid rice seedlings, and analyze the different mechanism by transcriptome sequencing.

## Materials and methods

### Plant growth conditions and drought stress treatments

Six rice varieties were collected from Shanghai Agrobiological Gene Center, China. Among them, HUHAN2B, CX181 and WPB106 were *japonica* varieties, while 9311, T1 and A3 were *indica* varieties. The embryonic callus induced from mature embryo of these rice varieties was treated with colchicine solution and obtained autotetraploid plants (Li and Lin et al. 2018). Seeds of six autotetraploid and diploid rice were sowed in plastic buckets. The soil was finely pulverized, and an equal amount of soil was filled in the buckets and used in the experiment. For each variety, 60 seeds of the tetraploid and diploid plants were sown with 30 seeds each in one bucket. When reaching four-leaf-age stage in growth chamber (30 °C, 14 h light/25°C,10 h dark cycle, humidity ~ 70%), irrigation was stopped for drought stress, and there was a 1–2 cm layer of water in the buckets under control conditions. After about three weeks, leaves were seriously curled and the tip of most leaves became yellow, then the plants were rewatered for the analysis of survival rate. The control plants were grown in the buckets under normal irrigation condition.

### Flow cytometry

Ten-day-old 2X and 4X rice seedlings were collected for ploidy analysis as described (Sun et al. 2022). The ploidy levels were measured by flow cytometry ‘BD Accuri C6’ (BD biosciences, USA).

### RNA-sequencing and data analysis

Total RNA was extracted from leaf under control and drought treatment conditions. For drought treatment, the leaves were sampled in the fifth day when the leaves start to curl. Three biological replicates of every tetraploid and diploid variety were prepared. RNA-seq libraries were constructed with TruSeq RNA kit from Illumina and sequenced on a HiSeq 2500 sequencer by Personal Bioinformatics Technology Co. Ltd (Shanghai, China). After filtering adapters and low-quality reads, the paired-end reads were mapped against the Nipponbare reference genome (IRGSP-1.0) using HISAT2. FPKM (fragments per kilobase per millon mapped reads) was then calculated to estimate the expression level of the genes. The differentially expressed genes (DEGs) were determined with the criteria of |log_2_(fold change)| ≥ 1 and *p*-value < 0.05 between two samples. GO (Gene Ontology; http://geneontology.org/) and KEGG (Kyoto Encyclopedia of Genes and Genomes, http://www.kegg.jp/) enrichment of DEGs were implemented by hypergeometric tests in which *p*-value is calculated and adjusted as q-value. GO and KEGG terms with q < 0.05 were considered significantly enriched. Heatmap is plotted using heatmap tools in the genescloud platform (https://www.genescloud.cn). The protein-protein interaction analysis was performed in STRING database (https://string-db.org/) (Szklarczyk et al. 2019). Based on reference genome, the mapped reads were assembled and spliced using the software StringTie (http://ccb.jhu.edu/software/stringtie/), and compared with known transcripts to obtain new transcripts without annotation information. Principal Component Analysis (PCA) was analyzed by EIGENSOFT (version 6.1.4) with default parameters.

### Co‑expression network module construction using association analysis module and physiological indexes

Weighted gene co-expression network analysis (WGCNA) was used to find clusters (modules) of highly correlated genes and relating modules to 4X rice seedlings of WPB106, HUHAN2B, 9311 and T1 subjected to drought and after rewatering. The method in “WGCNA” software package of Rv3.5.1 was followed. The exportNetworkToCytoscape function was used to export the module of interest to Cytoscape and vaisualize the network (Shannon et al. 2003).

### RNA isolation and qRT-PCR

Total RNA was extracted from leaf under control and drought treatment conditions. For drought treatment, the leaves were sampled in the fifth day when the leaves start to curl. Three biological replicates of every tetraploid and diploid variety were prepared. Total RNA was isolated from rice leaves using TRNzol reagent (TIANGEN, China). cDNA templates were synthesized using Superscript II reverse transcriptase (Transgen, China) according to the manufacturer’s instructions. Real-time quantitative RT-PCR was performed on a CFX96 Real-Time PCR system (Bio-Rad, USA) using SYBR Premix Ex Taq (Transgen, China) according to the manufacturer’s protocol. Rice *Actin1* (No. AY212324) gene was used as the endogenous control. The relative quantization method (△CT) was used to evaluate quantitative variation of replicates examined. The reaction mixture contained 10 µL of SYBR real-time PCR premixture, 0.5 µL cDNA, 0.5 µL of each of the forward and reverse primers (10 µM), and 8.5 µL of PCR grade water in a final volume of 20 µL. The following reaction conditions were applied: 2 min at 95℃, 40 cycles of 15 s at 95℃ and 30 s at 60℃. The primers are listed in Supplementary Material 1. Additional file 2: Table S1.

### Determination of physiological indices related to stress

At the four-leaf stage, the leaves began to curl after drought stress for 7 d. Leaves were sampled and performed a biochemical analysis of reactive oxygen species (ROS). H_2_O_2_ contents were determined following the manufacturer’s instructions as described previously (Rao et al. 2000). The activity of POD (peroxidase isozyme) and SOD (superoxide dismutase), GSH (glutathione), CAT (catalase), soluble protein and MDA (malondialdehyde) contents were determined according to the manufacturer’s instructions (Nanjing Institute of Bioengineering, Jiangsu, China). The photosynthetic rates were measured with the MIC-100 (Masa International Corporation, Japan) for 5 d following 15% PEG6000 treatment (from 9:30 a.m. to 10:30 a.m.) (Tanaka et al. 2022). Independent t-tests were applied to detect any significant differences of measured physiological traits between treatments with three replicates.

## Results

### Cell DNA content detection and leaf cytological observation

The autotetraploid (4X) rice arose from chromosome doubling of the diploid donor (2X) rice varieties, three *japonica* varieties HUHAN2B, CX181 and WPB106, and three *indica* varieties 9311, T1 and A3. Cellular DNA content of 2X and 4X rice seedlings were measured by flow cytometry. In six varieties, the channels with the standard peak of tetraploids all showed about double channels of their corresponding diploid lines (Supplementary Material 2 Additional file 1: Fig. S1). It indicated that the autotetraploids had nearly twice the DNA content as their donor diploids. Further observation revealed that the stomatal length and width of the tetraploids were significantly more extensive than those of the diploids (Supplementary Materia 3A Additional file 1: Fig. S2A). The stomatal density in diploids was about twice that of tetraploids (Supplementary Material 3B Additional file 1: Fig. S2B). The chloroplast number per stomatal guard cell in the tetraploids was higher than in the diploids (Supplementary Material 3C Additional file 1: Fig. S2C). Among them, *indica* tetraploids had about twice chloroplasts that of their diploid donors. The results indicated that 4X lines of six varieties originated from chromosome doubling of their 2X donor varieties.

### Drought stress phenotypic traits among diploid and autotetraploid rice varieties

**Fig. 1:**
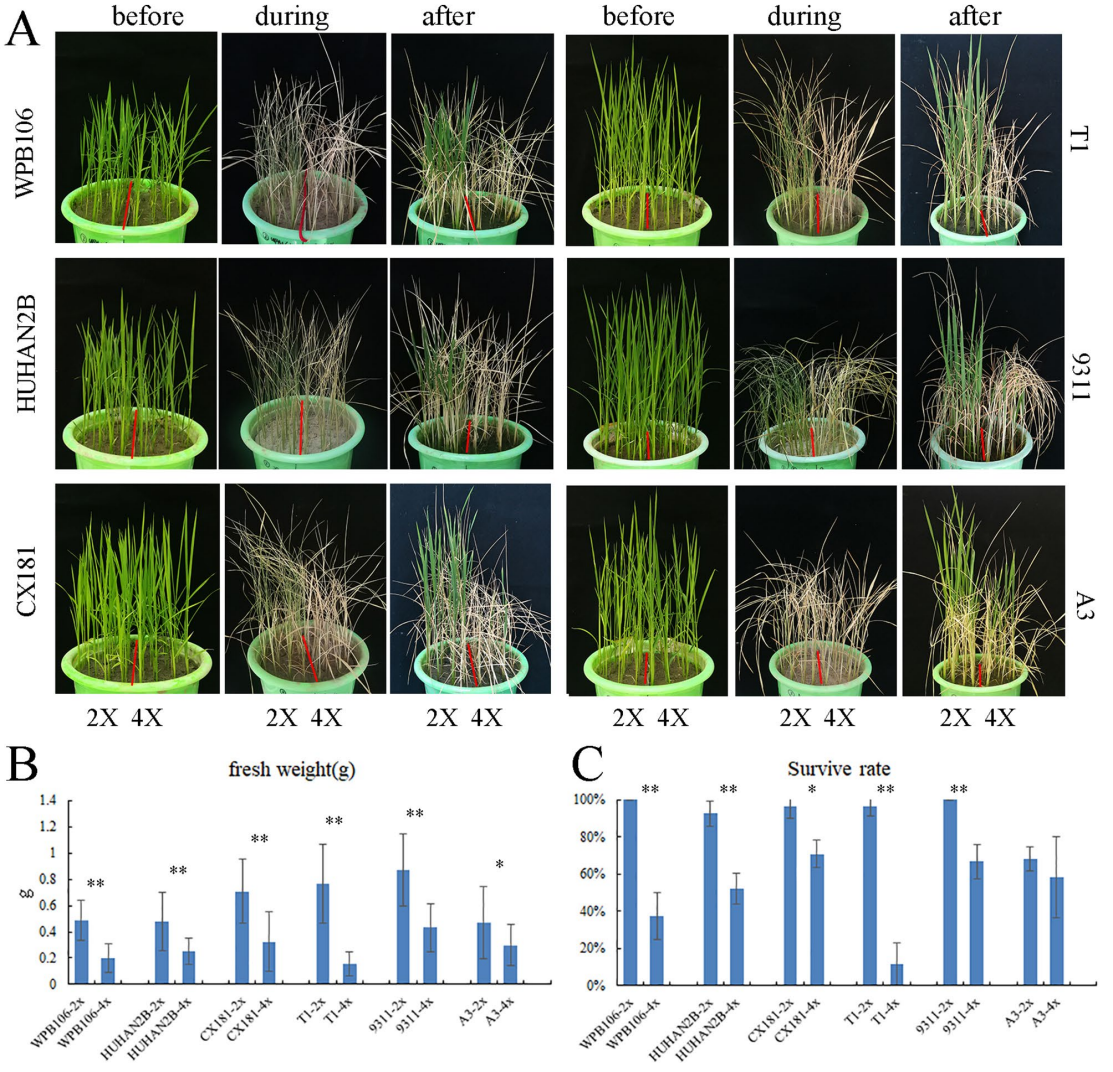
Drought tolerance between diploid and autotetraploid plants. Phenotypic changes of 2X and 4X of six varieties under drought stress (**A**). The plants grew in bucket until four-leaf stage (before). Irrigation stoped for about two weeks (during) and then rewatered 5 days (after). The fresh weight (**B**) and the survival rate (**C**) for soil drought stress. Error bars represent the three biological replicates (*P < 0.05, t test; **P < 0.01, t test). Significance analysis indicated the comparison between 2X and 4X of the same variety

In order to compare the drought tolerance between 2X and 4X plants, the drought stress was conducted in the plastic bucket with soil. The seedlings were grown in bucket until four-leaf stage. Then watering was stopped and leaves began to curl after 7 d. The leaves of tetraploids curled severely and scorched ahead of diploids (Fig. 1A). After about two weeks, plants were rewatered. After recovery for 5 days, the survival rate was counted. The results revealed that the survival rate of autotetraploids is significantly lower than that of diploids, except for A3 variety between A3-2X and A3-4X (Fig. 1C). Moreover, the fresh weight of all 4X lines was significantly lower than that of all 2X lines. It indicated that chromosome doubling of diploid rice weakened their drought tolerance.

### Effect of biochemical traits after osmotic stress

**Fig. 2:**
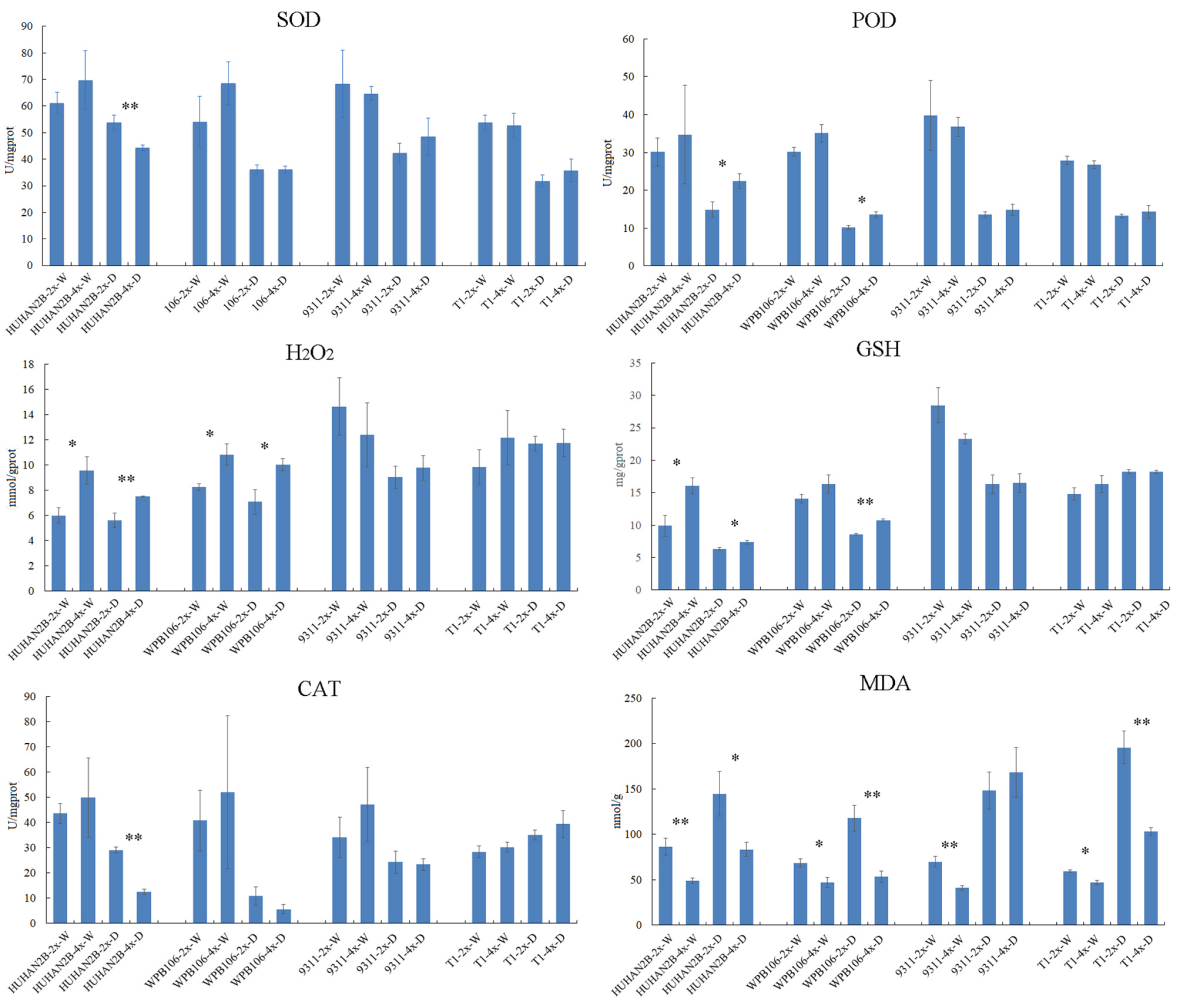
Physiological characteristics between diploid and autotetraploid rice plants under drought stress. The plants at four-leaf stage were growth under normal condition and treatment with 15% PEG6000. SOD, POD, H_2_O_2_, GSH, CAT and MDA were detected at third day after osmotic stress. Error bars represent the three biological replicates (*P < 0.05, t test; **P < 0.01, t test). Significance analysis indicated the comparison between 2X and 4X of the same variety. W, normal condition; D, osmotic stress. 2X, diploid; 4X, autotetraploid

Plant reacts to drought stress by producing excess reactive oxygen species (ROS), leading to cellular damage. Antioxidant contents and the activities of ROS scavenging enzymes are used to evaluate the response to drought stress. Figure 2 showed that the activity of SOD, POD or CAT in *indica* 4X lines is not significantly different compared with their corresponding 2X lines under drought stress, as well as the content of H_2_O_2_ and GSH (Fig. 2). In *japonica* lines, however, SOD and CAT activities of HUHAN2B autotetraploid are lower than its corresponding diploid lines under drought stress, whereas the POD activity in *japonica* 4X lines are higher than their corresponding 2X lines under drought stress, as well as H_2_O_2_ and GSH content (Fig. 2). The common characteristics between *indica* and *japonica* lines is that the MDA content in autotetraploid lines is significantly lower than their corresponding diploid lines under control and drought conditions, except for in 9311 lines under drought stress (Fig. 2). These indicated that the variation of ROS was not the reason for the decrease of drought tolerance in autotetraploid lines.

### Effect of ployploidization on photosynthetic rates after osmotic stress

**Fig. 3:**
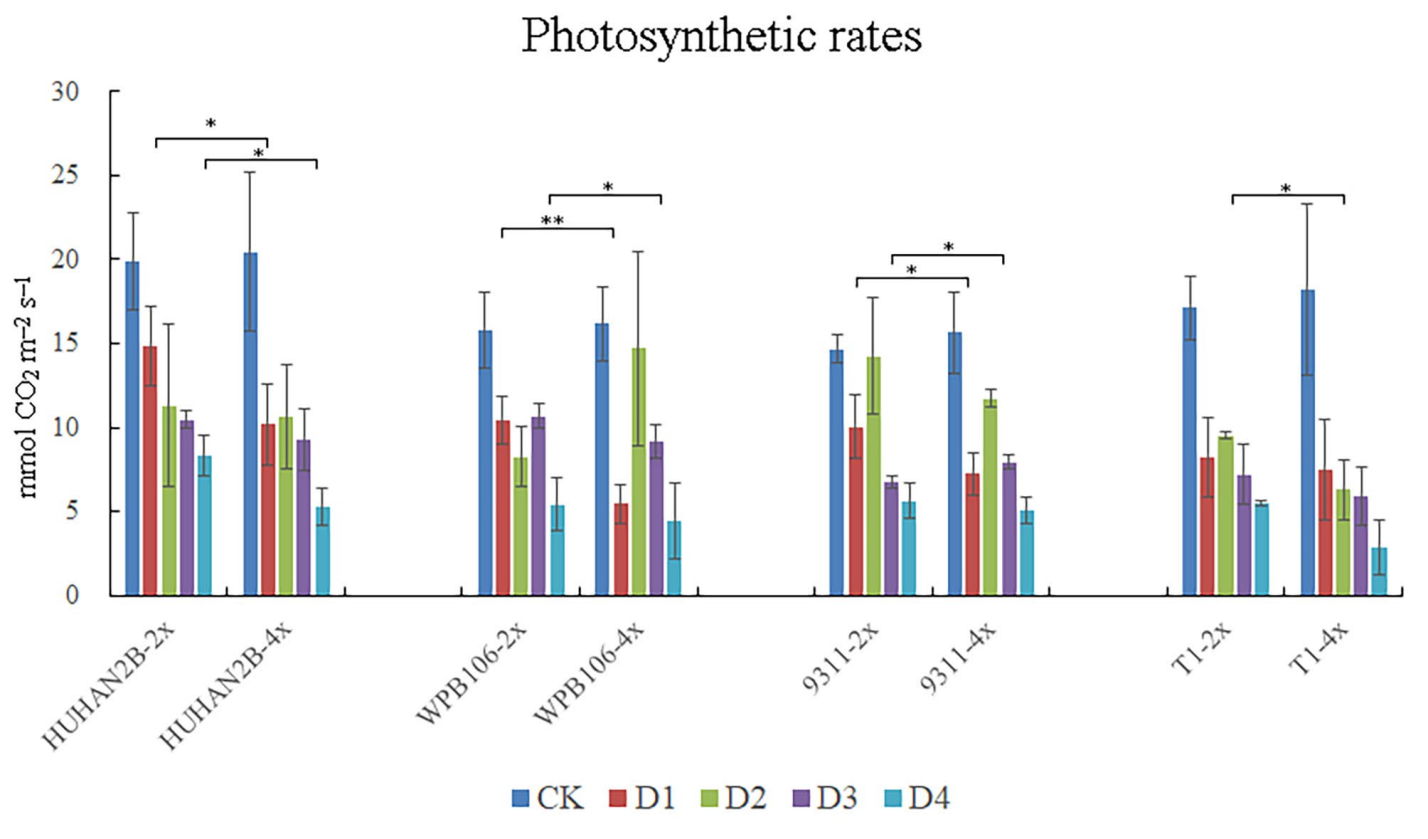
Photosynthetic rates between diploid and autotetraploid plants after osmotic stress. The plants at four-leaf stage were growth under normal condition and treatment with 15% PEG6000 at about 10 a.m. Photosynthetic rate was measured by MIC-100 at about 10 a.m. every day. Error bars represent the five biological replicates (*P < 0.05, t test; **P < 0.01, t test). Significance analysis indicated the comparison between 2X and 4X of the same variety. CK, normal condition. D1, 1 d for osmotic stress. D2, 2 d for osmotic stress. D3, 3 d for osmotic stress. D4, 4 d for osmotic stress

Rice under water deficit stress exhibits decline in photosynthetic rate. Under control condition, there is no significant difference in photosynthetic rate between 2X and 4X lines of all varieties (Fig. 3). However, decline in photosynthetic rate of 4X lines is sharper than of its corresponding 2X lines at different time points under osmotic stress for different varieties. After 1 d under osmotic stress, the photosynthetic rate of HUHAN2B-4X, WPB106-4X and 9311-4X decreased more dramatically than of its donors, and the rate of T1-4X descended significantly after 2 d (Fig. 3). Finally, the photosynthetic rate of all 4X lines was lower than of their corresponding 2X lines. The results indicated that the decline of drought tolerance was due to the decline of photosynthetic rate.

### Identification of differential gene expression under drought treatment

**Fig. 4:**
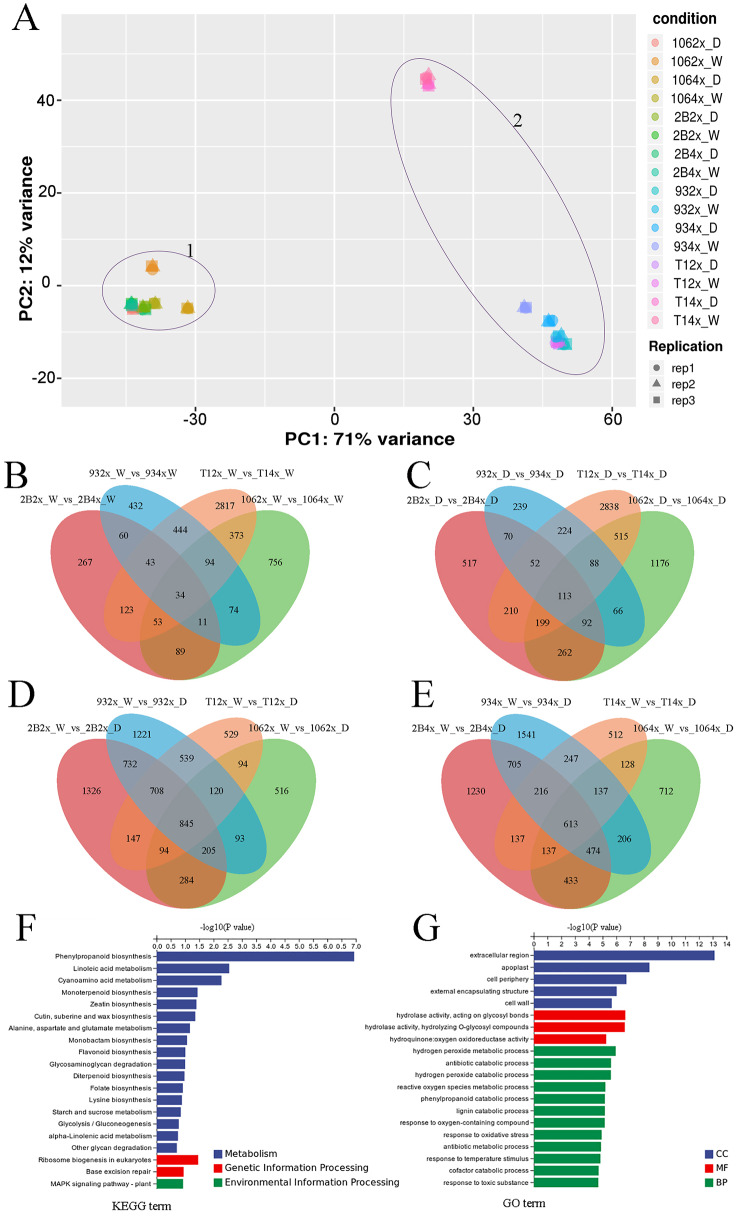
Genome-wide gene expression between diploid and autotetraploid plants under control and drought stress. (**A**) Results of principal component analysis for total 2X and 4X lines based on RNA resequencing data. PC, principal component. (**B**) Venn diagram of 2X and 4X in control condition. (**C**) Venn diagram of 2x and 4x in drought stress. (**D**) Venn diagram of 2X between control condition and drought condition. (**E**) Venn diagram of 4X between control condition and drought condition. 106 indicated WBP106, 2B indicated HuHan2B, 93 indicated 9311 and T1 indicated T1 variety. 2X, diploid; 4X, tetraploid. W, normal condition; D, drought condition. (**F**) Kyoto Encyclopedia of Genes and Genomes (KEGG) enrichment analysis of common DEGs based on B-E. (**G**) Gene ontology (GO) enrichment analysis of common DEGs based on B-E. MF, Molecular Function; CC, Cell Component; BP, Biological Process

To investigate the intrinsic differences between diploid and autotetraploid of different varieties under drought stress, gene expression levels of four varieties were evaluated with RNA-sequencing. Transcriptomic analysis detected 2251, 4341, 4463 and 3076 differential expression genes (DEGs) from drought stress in the 2X lines of WPB106, HUHAN2B, 9311 and T1, as well as 2840, 3945, 4139 and 2127 in the corresponding 4X lines, respectively. The expression levels of several DEGs were checked by RT-qPCR. The results confirmed the expression change of most of the selected DEGs before and after drought treatment in four varieties (Supplementary Material 4 Additional file 1: Fig. S3). DEGs statistics indicated that there was obvious difference in the number of DEGs responding to drought stress among different varieties, but there was no obvious difference between the corresponding diploid and autotetraploid. *japonica* varieties (cluster 1) and *indica* varieties (cluster 2) are well-separated by the PCA, and 2X lines and 4X lines of each variety was not separated by the PCA, except for T1 4X lines (Fig. 4A). Clustering results showed that all DEGs were divided into two clusters under drought condition and control condition, except for T1-4X in drought condition (Supplementary Material 5A Fig. S4A). Venn maps of DEGs from four different kinds of compare analysis showed that the number of common DEGs between 2X and 4X under same conditions is less than that of same ploidy under different conditions (Fig. 4B and E). It suggested that there was a common mechanism between different varieties in response to drought stress. However, there were obvious distinctions in the number of unique DEGs among different varieties either under the control condition or the drought condition (Fig. 4 and Supplementary Material 5A Fig. S4A). It indicated that the wide variation of gene expression was caused by difference of varieties, not by ploidy.

To examine the function of the common DEGs identified between 2X and 4X of the different varieties, both KEGG (Fig. 4F) and GO enrichment analyses (Fig. 4G) were performed. The union of the common DEGs included the common DEGs in shoots between 2X and 4X under control condition (Fig. 4B), under drought stress (Fig. 4C), 2X (Fig. 4D) and 4X (Fig. 4E) between control condition and drought condition, respectively. The top enriched KEGG terms in metabolism included phenylpropanoid biosynthesis, linoleic acid metabolism, cyanoamino acid metabolism and other secondary metabolism. In genetic information processing, the genes of ribosome biogenesis in eukaryotes and base excision repair were also significantly enriched. Besides, MAPK signaling pathway-plant was one of the most enriched KEGG terms. GO analysis revealed that extracellular region was enriched in cell component term, hydrogen peroxide metabolic process and response to oxidative stress in biological process term. The results suggested that there was common mechanism to respond to drought stress between 2X and 4X plants of different varieties.

### Gene expression modules of photosynthesis related to drought tolerance

**Fig. 5:**
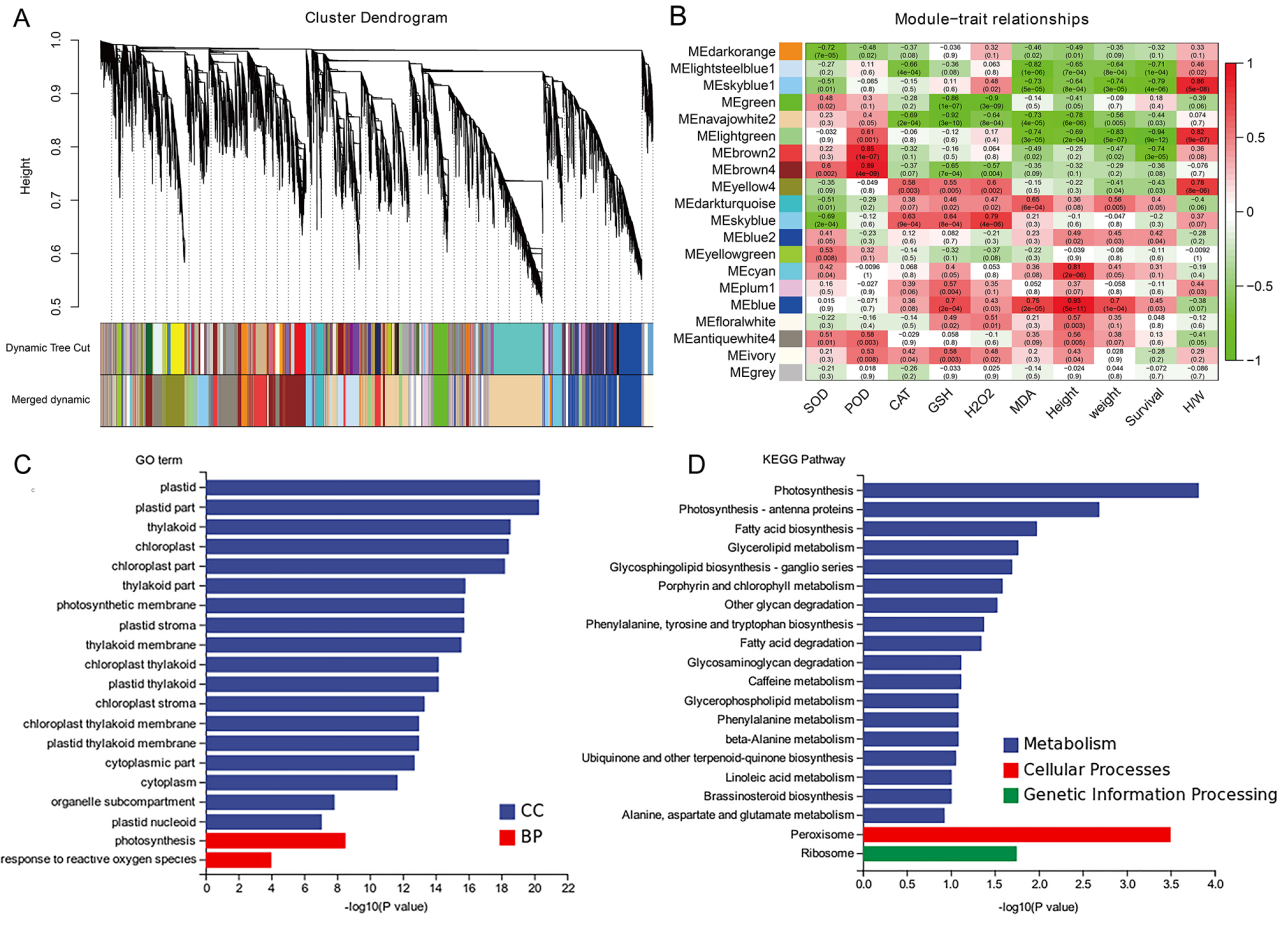
The co-expression analysis of diploid and autotetraploid plants under drought stress. (**A**) Analyze the co-expression gene network under drought stress by WGCNA, and the genes were clustered into different co-expression modules with different colors. (**B**) Correlation analysis was carried out between the co-expression modules of various genes and physiological indexes, plant phenotype under drought conditions. The number above the heat map represents Pearson correlation coefficient (r) and *p* value. SOD, POD and CAT indicated the activity of superoxide dismutase, peroxidase and catalase, respectively. GSH, H_2_O_2_ and MDA indicated the content of glutathione, H_2_O_2_ and malondialdehyde, respectively. Height, Weight, Survival and H/W indicated plant height, fresh weight, survival rate and the rate of height and weight under drought condition. (**C**) Gene ontology (GO) enrichment analysis of MElightgreen module. CC, Cell Component; BP, Biological Process. (D) Kyoto Encyclopedia of Genes and Genomes (KEGG) enrichment analysis of MElightgreen module

To investigate key genes and pathways involved in the process of drought treatment, we performed weighted gene co-expression network analysis (WGCNA) to identify the expression module of genes responding to drought stress in the 4X lines of WPB106, HUHAN2B, 9311 and T1. The co-expression network analysis showed that these genes were divided into 20 co-expression modules (Fig. 5A). The correlation analysis of the modules with physiological indicators and plant phenotype revealed that MElightgreen module was significantly correlated with survival rate with the highest absolute value of correlation coefficient (r) and the lowest *p* value (Fig. 5B). Additionally, the module was also correlated with POD, MDA, plant height and fresh weight. The results suggest that the module negatively regulates the survival rate and influence the plant growth under drought stress.

The MElightgreen module contained 863 genes (Supplementary Material 6 Additional file 2: Table S2). Both GO and KEGG enrichment analyses were performed. The results revealed that the top enriched GO terms were mainly involved in photosynthesis system in cell component and biological process terms (Fig. 5C). Similarly, the top enriched KEGG pathways were also photosynthesis in metabolism term (Fig. 5D). Additionally, these genes involved in fatty acid metabolism, amino acid metabolism and other secondary metabolism were also enriched. Peroxisome and ribosome also indicated that the module participated the process of response to reactive oxygen species and RNA translation. It can be speculated that the variation of gene expression in photosynthesis system is the primary impact factor for the decrease of drought tolerance.

**Fig. 6:**
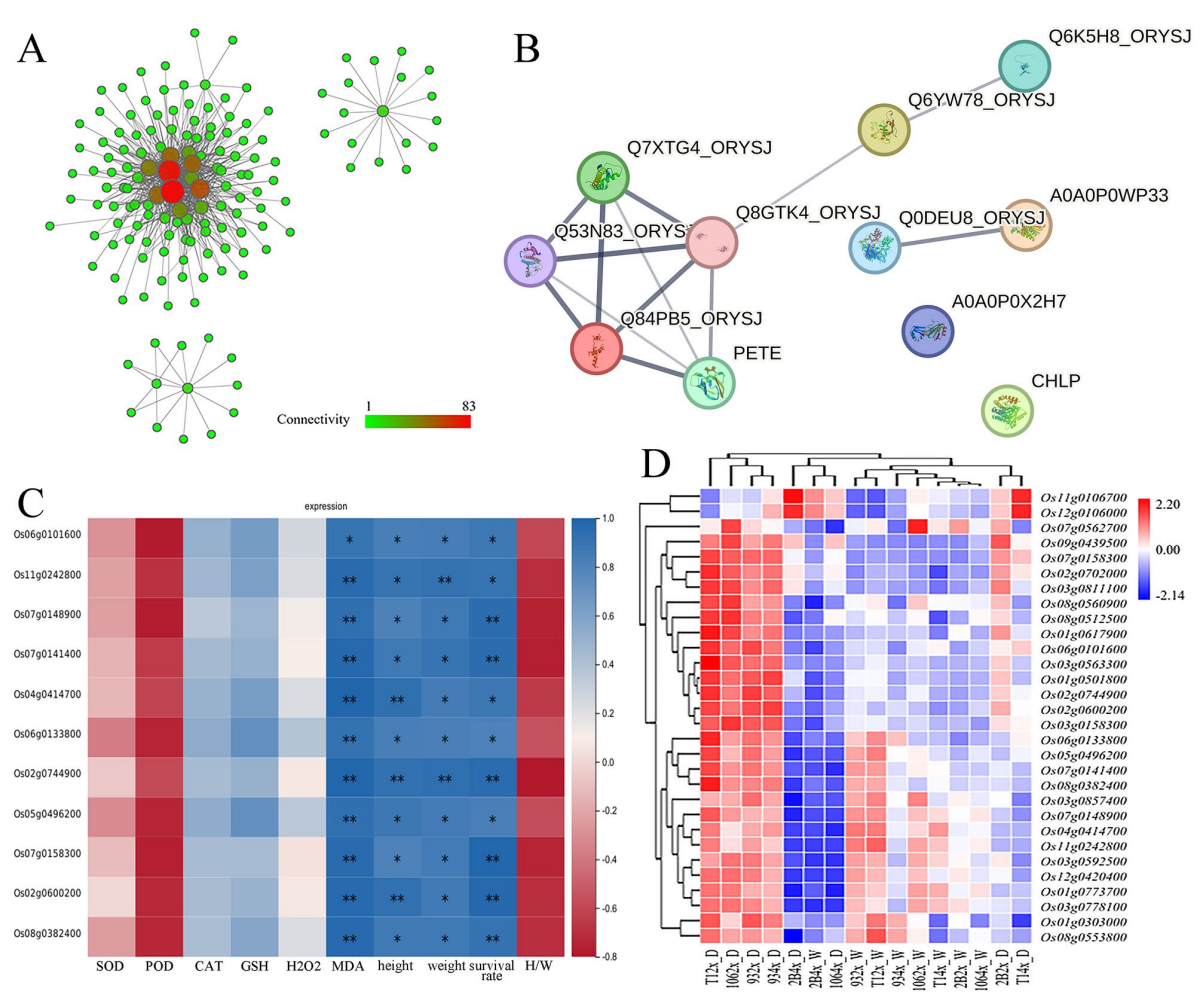
The expression analysis of photosynthesis system under drought stress. (**A**) The weighted network diagram of MElightgreen module. The size and color of the circle represent the number of relationships with other genes. (**B**) The protein-protein interaction (PPI) network of eleven hub genes using the STRING database. Colored nodes: query proteins and first shell of interactors. white nodes: second shell of interactors filled nodes: some 3D structure is known or predicted. The line thickness represents edge confidence. Q53N83_ORYSJ (Os11g0242800), Q7XTG4_ORYSJ (Os04g0414700), Q8GTK4_ORYSJ (Os07g0141400), A0A0P0 × 2H7 (Os07g0158300), Q0DEU8_ORYSJ (Os06g0133800), PETE (Os06g0101600), Q6K5H8_ORYSJ (Os02g0600200), CHLP (Os02g0744900), Q6YW78_ORYSJ (Os08g0382400), A0A0P0WP33 (Os05g0496200), Q84PB5_ORYSJ (Os07g0148900). (**C**) The correlation heatmap between gene expression and physiological indexes, plant phenotype under drought conditions. Color shades indicate correlation; * for *p* < 0.05, ** for *p* < 0.01. (**D**) Interactive heatmap of gene expression in photosynthesis system. 106 indicated WBP106, 2B indicated HuHan2B, 93 indicated 9311 and T1 indicated T1 variety. 2x, diploid; 4x, tetraploid. W, normal condition; D, drought condition

In order to identify hub genes in the MElightgreen module, the data (weight > 0.25) was applied to draw a weighted network graph with parameters set to a soft threshold range of 5-1000. This approach yielded eleven node genes (Fig. 6A). The eleven genes belong to Chloroplast component (GO:0009507) and eight genes belong to Photosynthesis (GO:0015979) term (Supplementary Material 7 Table S3). These genes participate photosystem II assembly, light harvesting in photosystem I and reductive pentose-phosphate cycle. Further protein-protein interaction (PPI) analysis revealed that there was interaction in proteins encoded by nine of the eleven genes, including seven proteins with tight interaction (Fig. 6B). Meanwhile, the expression of eleven genes under drought stress was negatively correlated with survival rate, height, fresh weight and MDA (Fig. 6C). To gain a deeper understanding of gene expression in the photosynthetic system, 30 genes located in the network of eleven node genes were selected for observation, which belong to GO:0015979 (photosynthesis) and GO:0009507 (chloroplast) in the MElightgreen module. The results showed that the expression of most of genes related to photosynthesis in diploid plants was higher than in 4X lines, except for 9311(Fig. 6D). In addition, the expression was positively correlated with POD and the ratio of height and weight (Fig. 6D). POD participates in photorespiration and oxidizes glycolic acid, a by-product of photosynthesis, to glyoxylic acid and hydrogen peroxide (Pan et al. 2020). Interactive heatmap of gene expression related with POD was constructed and showed that the genes were divided into three clusters. The gene expression of Cluster I and III under drought stress in diploid plants was lower than in autotetraploid plants, except for Huhan2B (Supplementary Material 5B Additional file 1: Fig. S4). The genes included 3-hydroxy-3-methylglutaryl-coenzyme A reductase (*Os09g0492700*) and glycolate oxidase (*Os04g0623500*). Glycolate oxidase (GLO) is an important enzyme in photorespiratory metabolism, and its expression advance in autotetraploid implied the strengthening of photorespiratory. However, the gene expression of Cluster II under drought stress in diploid plants was higher than in autotetraploid plants, except for 9311. The genes included peroxidase (*Os08g0522400*) and superoxide dismutase (*Os06g0115400*) (Supplementary Material 5B Additional file 1: Fig. S4). The results indicated that the substantial disruption of photosynthesis-related genes in tetraploid plants under drought stress serves as the principal explanation for the decrease of drought resistance in autotetraploid plants, compared with diploid.

### New transcripts from autotetraploid under drought condition

**Fig. 7:**
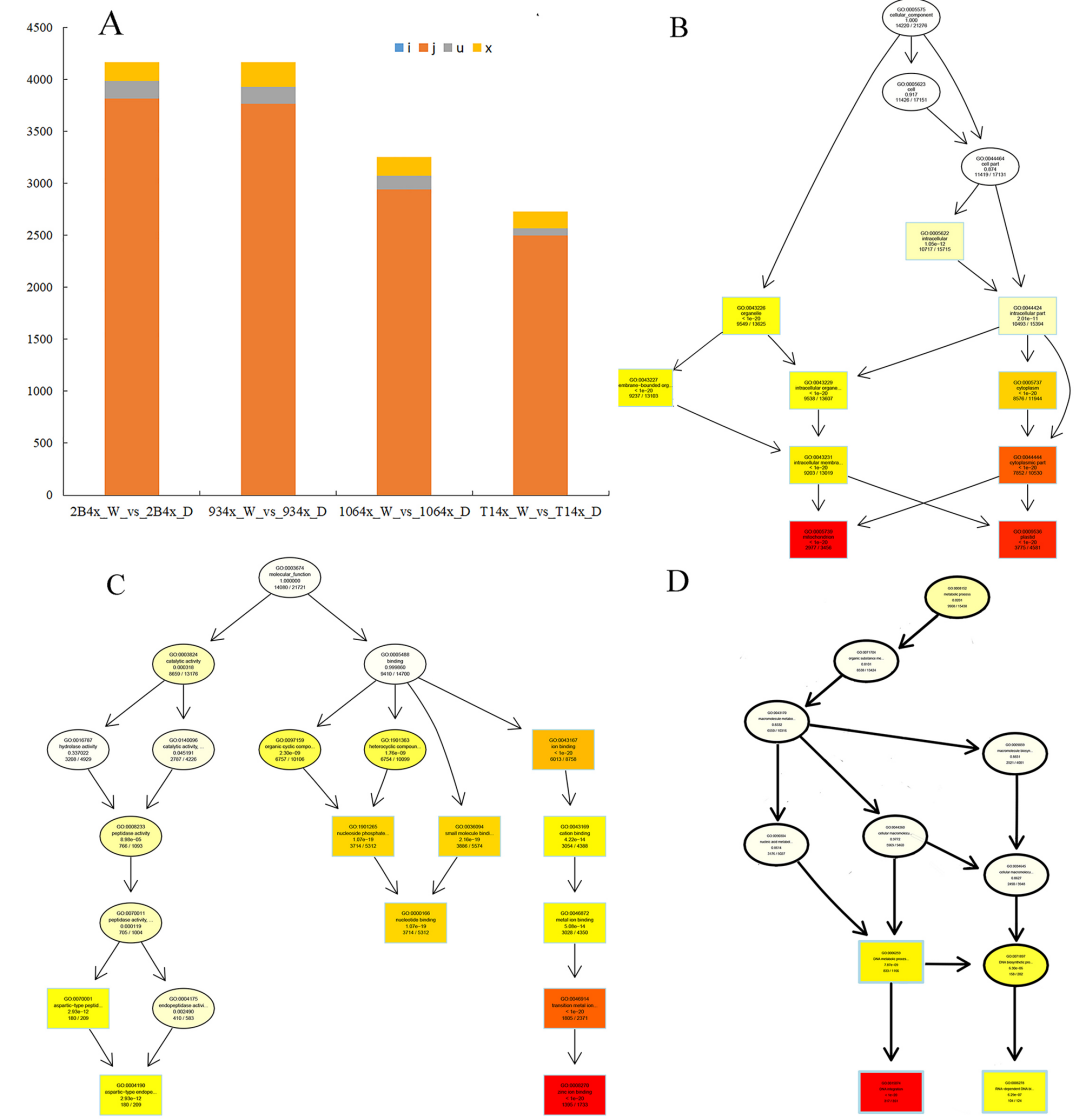
The representative terms in GO enrichment analysis of new transcripts from autotetraploid plants. (**A**) The mitochondrion and plastid are two of top ten terms in Cell Component terms in tetraploid plants. (**B**) The top ten terms in Molecular Function terms in tetraploid plants. The darker the color is, the more significant the GO term is, and the color from light to deep is colorless-light yellow-deep yellow-red

Isoform sequencing technology yields long reads without the aid of assembly and help to identify new transcripts in autotetraploid. Based on rice reference genome, the software StringTie was used to assemble and splice the mapped reads. Compared them with known transcripts, the transcripts without annotation information were identified as new transcripts with Class Code “j”, “i”, “u” and “x“(Szklarczyk et al. 2019). Among them, “x” was antisense transcripts of known transcripts. Therefore, functional annotation was performed on all new transcripts. Compared with autotetraploid plants under normal and drought condition, the numbers of the different new transcripts range from 2730 to 4170 and are no significant different between *indica* and *japonica* (Fig. 7A). The antisense transcripts (class code “x”) account for about 5% among four varieties. GO enrichment analysis of new transcripts from autotetraploids revealed that mitochondrion and plastid of cell component were top two items. The results suggested that the integrality of mitochondrion (GO: 0005739) and plastid (GO: 0009536) was influenced by ploidy (Fig. 7B). In the molecule function item, nucleotide (GO: 0000166) and metal ion binding (GO: 0008270) were significantly enriched (Fig. 7B). Moreover, DNA integration (GO: 0015074) and RNA-dependent DNA biosynthetic process (GO: 0006278) in Biological Process item were significantly enriched (Fig. 7C). The results also suggested that chromosome duplication influenced DNA integration and RNA-dependent DNA biosynthetic process, finally influencing cell tolerance to stress.

**Fig. 8:**
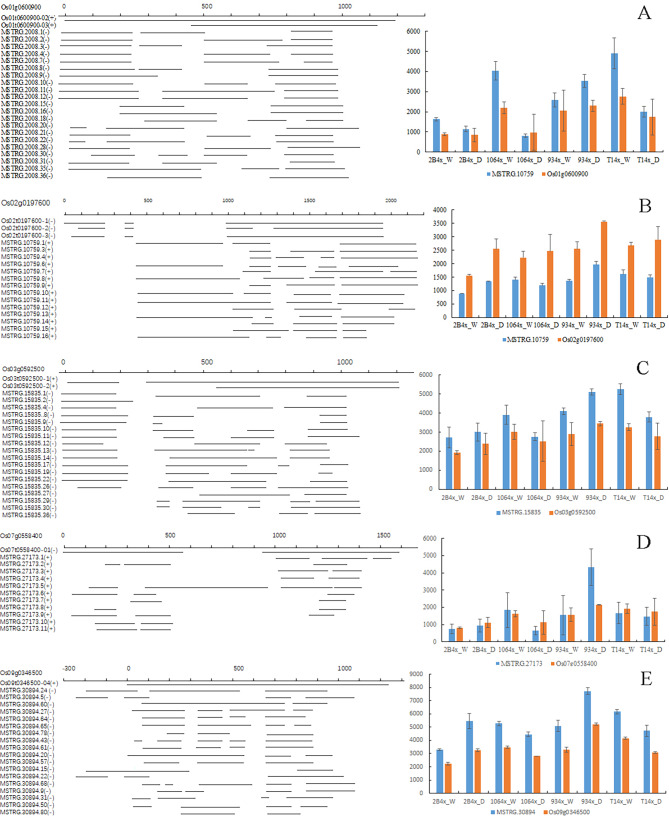
New antisense transcript isoforms and expression of five chlorophyll A-B binding genes from autotetraploids. (**A**) *Os01g0600900*. (**B**) *Os02g0197600*. (**C**) *Os03g0592500*. (**D**) *Os07g0558400*. (**C**) *Os09g0346500*. Left, transcripts of mRNA (Os) according to gene annotations in the reference genome and new identified transcripts (MSTRG) in autotetraploids. Right, transcript expression levels of genes and total new transcripts. + indicates positive strand on chromosome, and - indicates negative strand. Each unit of genome length is 100 bp. 0 represents the transcription start sites of genes

Chloroplasts perform photosynthesis based on proper assembly of proteins and integrality of plastid. Plastid of cell component was one of top enrichment items (Fig. 7B), and photosynthetic rate of all autotetraploid varieties significantly decreased compared with their diploid donors (Fig. 3). The transcripts MSTRG.2008, MSTRG.10,759, MSTRG.15,835 and MSTRG.27,173 were mapped onto antisense strands of *Os01g0600900*, *Os02g0197600*, *Os03g0592500*, *Os07g0558400* and *Os09g0346500* genes, which all encoded chlorophyll A-B binding proteins (Fig. 8). Cumulative quantity of new transcripts was higher than the gene expression of chlorophyll A-B binding proteins, except for *Os02g0197600* (Fig. 8B). Additionally, the aberrant mRNA transcripts often produce aberrant, truncated or non-functioning proteins. On the same strand, the transcript of MSTRG.31,903 mapped onto *Os09g0532000*, a Stay-Green (*OsSGR*) gene encoding the chlorophyll-degrading Mg^2+^-dechelatase (Shin et al. 2020). The transcript of MSTRG.11,831 matched *Os02g0538000*, encoding Threonyl-tRNA synthetase (TSV2) in chloroplast development at the early leaf stage (Lin et al. 2021). MSTRG.14,063, MSTRG.30,524 and MSTRG.904 matched *Os03g0163300*, *Os09g0249900* and *Os12g0292900* involved in chloroplast electron transport chain and redox state, respectively (Chou et al. 2012; Buchanan 2016; Muller-Schussele et al. 2020) (Supplementary Material 8 Additional file 2: Table S4). Under drought stress, these aberrant mRNA transcripts ultimately cause chloroplast damage and weaken drought tolerance.

## Discussion

The advantage of polyploids in plant growth, yield and environmental fitness is a great temptation for crop breeders to create new polyploids in modern crop trait improvement (Paterson 2005; Van de Peer et al. 2017). Autotetraploid rice from artificial genome duplication is used as a germplasm to evaluate the environmental adaptability. Until now, autotetraploids of more than four varieties showed superior resistance to salt or alkali stress compared with its diploids (Wu et al. 2013; Wang et al. 2022a, b; Zhang et al. 2022). Can tetraploid rice also improve the drought tolerance? Our results showed that autotetraploids from six *indica* and *japonica* varieties all impaired the drought tolerance in the seedling stage (Fig. 1). In adult stage, the biomass, plant height, grain weight, tiller number did not coincident among different varieties under normal and drought condition, but the yield per plant and seeding rate of 4X varieties were lower than of its 2X varieties (Supplementary Material 9 Fig. S5). It suggested that autotetraploids might impair the drought tolerance in the whole growth stage. However, the survival rate of autotetraploids is not significantly different between A3-2X and A3-4X although their fresh weights exhibit a significant difference (Fig. 1C). It suggested that the genetic background of diploids might influence the trait of its tetraploids under environmental stress. Additionally, stomata play important roles in the conductance, transpiration and photosynthetic traits of plants. It has been suggested that rice with high stomatal density and small stomatal size displayed faster stomatal closure and can be chosen to tackle drought stresses (Caine et al. 2023). In this study, lower stomatal density in tetraploids cannot compensate for the effect of bigger stomatal size under drought stress compared with diploids (Additional file 1: Fig. S2). It suggests that the stomatal structure in tetraploid is favorable for plant growth under normal conditions, but unfavorable for tolerance to drought stress.

What are the factors that determine the decline in drought resistance in tetraploids? The ability of ROS scavenging is an important guarantee for plants to overcome drought stress. The difference of SOD, POD, CAT, GSH activity and H_2_O_2_ in *indica* 4X lines is not significant, and the change tendency is not consistent in different *japonica* 4X lines compared with their corresponding 2X lines under drought stress (Fig. 2). The results indicated that the level of ROS is not the determining factor of the decrease of drought tolerance in autotetraploid lines. MDA is used to predict the oxidative lipid injury whose concentration varies in response to abiotic stress. In this study, MDA content in most of 4X lines is significantly lower than their corresponding 2X lines under control and drought conditions (Fig. 2). Yang et al. (2014) also found lower MDA content in SP-4X and 630-4X, indicating high integrality and low peroxidation level of cell membranes in the autotetraploid rice under drought stress (Yang et al. 2014; Tu et al. 2014). However, only fitness of cell membranes is not enough to maintain the drought stress. In our study, the photosynthetic rate of all 4X lines decreased more under osmotic stress, compared with its corresponding 2X lines (Fig. 3). Therefore, we speculate that the dramatic decrease in photosynthetic rate is the major limitation to overcome drought stress in 4X lines compared with their corresponding 2X lines. Howbeit, Yang et al. (2014) thought that the chromosome-doubling enhanced rice photochemical efficiency and photoinhibition tolerance under drought stress through chloroplast fluorescence detection (Yang et al. 2014). It is possible that difference in 2X donor has an important impact on its corresponding tetraploids, although they did not described the variety subspecies and phenotype under drought stress.

To further explore the reasons for the weakening of drought resistance of autotetraploids, we performed transcriptome-sequencing analysis of 2X and 4X of four varieties under normal and drought condition. Venn maps of DEGs showed that the number of common DEGs between ploidies was smaller than that between varieties, and chromosome doubling reduced the number of common DEGs under drought stress (Fig. 4B and E). It suggested that chromosome doubling further expanded the differences between varieties, especially under drought stress. In previous study, chromosome doubling is considered to have detrimental effects on fertility and fitness owing to genomic instability, mitotic and meiotic abnormalities, and gene expression and epigenetic confusion (Van de Peer et al. 2017). Wang et al. (2021) found that polyploidy induces DNA hypomethylation and salt stress-induced hypermethylation to repress proximal TEs and/or TE-associated stress-responsive genes in 4X of two *japonica* varieties (Wang et al. 2021). Autopolyploids lead to a higher number of accessible chromatin regions (ACRs) in euchromatin, and modulate chromatin signatures and transcriptional profiling (Zhou et al. 2021). The results suggested that chromosome doubling magnified genomic instability, epigenetic variability and chromatin accessibility. The changes may be a double-edged sword that can have negative effects under certain conditions, including drought stress. Additionally, we found that correlation might be highly influenced by phenotype difference between varieties, as this difference is larger than ploidy difference (Figs. 1 and 2). Therefore, we think genetic material of every variety is decisive for phenotype and ploidy effect is a dose-response relationship. However, it takes a process to go from quantitative to qualitative.

WGCNA analysis reveals that genes associated with the photosynthetic system play a crucial role. MElightgreen module was significantly correlated with survival rate, POD, MDA, plant height and fresh weight (Fig. 5B). In the MElightgreen module, genes associated with photosynthesis system were not only top enrichment item (Fig. 5C and D), but also key genes to draw a weighted network graph (Fig. 6A). Moreover, the expression of most of genes related with photosynthesis in diploid plants was higher than in autotetraploid plants, except for 9311(Fig. 6C). Chromosome doubling may influence gene transcription. New transcripts analysis from autotetraploid revealed that the gene transcription related with mitochondrion and plastid of cell component was influenced most significantly (Fig. 7B). There were high abundant antisense transcripts localized at five genes encoding chlorophyll A-B binding proteins (Fig. 8). Additionally, the aberrant mRNA transcripts related with chloroplast metabolism and development were found (Additional file 2: Table S4). These antisense aberrant transcripts may disturb the normal mRNA transcript related with chloroplasts system, and aberrant proteins in chloroplasts may form inactive complexes that compete with functional complexes, may assemble into aggregates with non-function or toxicity, or may introduce harmful activities if mislocalized (Gardner et al. 2005). Among the six 4X rice lines, expression confusion caused by chromosome doubling damaged the plant development under drought stress.

Recently polyploidization is a new strategy developed to improve crop yield and abiotic stress resistance. It is also believed that tetraploid rice can improve salt and alkaline tolerance (Wang et al. 2022a, b; Zhang et al. 2022). However, autotetraploidization does not potentiate the tolerance of its diploid rice to drought stress in this study. The primary reason is the serious damage in gene expression of chloroplast system, protein function and photosynthetic rate under drought stress. Interestingly, we can find that not all autotetraploids have significantly decreased survival rates (Fig. 1C). Additionally, the change of POD activity and GSH content in *japonica* autotetraploid lines are not similar to *indica* lines under drought stress (Fig. 2). The expression pattern of most genes related with photosynthesis in 9311 lines was different from other lines (Fig. 6D). It suggested that the selection of donor genetic background of diploid is of great value for drought tolerance of its autotetraploid lines. Neo-tetraploid rice developed from crossing and directional selection of different autotetraploid rice lines (Yu et al. 2020) is possible to become a new tetraploid rice germplasm for abiotic stress breeding. However, it still needs a great deal of efforts for comprehensive improvement.

## Conclusion

In summary, autotetraploidy of rice does not potentiate the tolerance to drought stress in the seedling stage through the assay of drought tolerance among six varieties of *indica* and *japanica* rice. This was not due to differences in the capacity for reactive oxygen species production and scavenging betwee 2X and 4X varieties, but due to differences in photosynthetic rate. All 4X varieties had lower photosynthetic rates than their diploid donors. Further, transcriptomic analysis of 2X and 4X plants of four varieties under normal and drought condition showed that the wide variation of gene expression was caused by difference of varieties, not by chromosome ploidy. Weighted gene co-expression network analysis (WGCNA) and new transcripts analysis revealed that the severe interference of photosynthesis-related genes in tetraploid plants under drought stress is the primary reason for the decrease of drought tolerance in autotetraploid lines. The results indicated that chromosome doubling of diploid rice weakened their drought tolerance, primarily due to disorder of photosynthesis-related genes in tetraploid plants under drought stress. It is crucial to maintain tetraploid drought tolerance through chromosome doubling breeding in rice needs to start with the selection of parental varieties and screening evolution of multiple adversities.

### Electronic Supplementary Material

Below is the link to the electronic supplementary material.

Supplementary Material 1

Supplementary Material 2

Supplementary Material 3

Supplementary Material 4

Supplementary Material 5

Supplementary Material 6

Supplementary Material 7

Supplementary Material 8

Supplementary Material 9

Supplementary Material 10

## Data Availability

Sequence data that support the findings of this study have been deposited in the NCBI Sequence Read Archive (https://www.ncbi.nlm.nih.gov/sra/) under the accession number PRJNA1071349.

## Abbreviations

2X: diploid
4X: autotetraploid
WGCNA: weighted gene co-expression network analysis
JA: jasmonic acid
TEs: transposable elements
MDA: malondialdehyde
FPKM: fragments per kilobase per millon mapped reads
DEGs: differentially expressed genes
GO: Gene Ontology
KEGG: Kyoto Encyclopedia of Genes and Genomes
qRT-PCR: quantitative reverse transcription-polymerase chain reaction
ROS: reactive oxygen species
POD: peroxidase isozyme
SOD: superoxide dismutase
GSH: glutathione
CAT: catalase
MAPK: The mitogen activated protein kinases
