# Supplementary figures and images for: Autotetraploidy of rice does not potentiate the tolerance to drought stress in the seedling stage
Source: Rice (N Y). 2024 Jun 18;17:40. doi: 10.1186/s12284-024-00716-w (PMC11189374; doi:10.1186/s12284-024-00716-w)

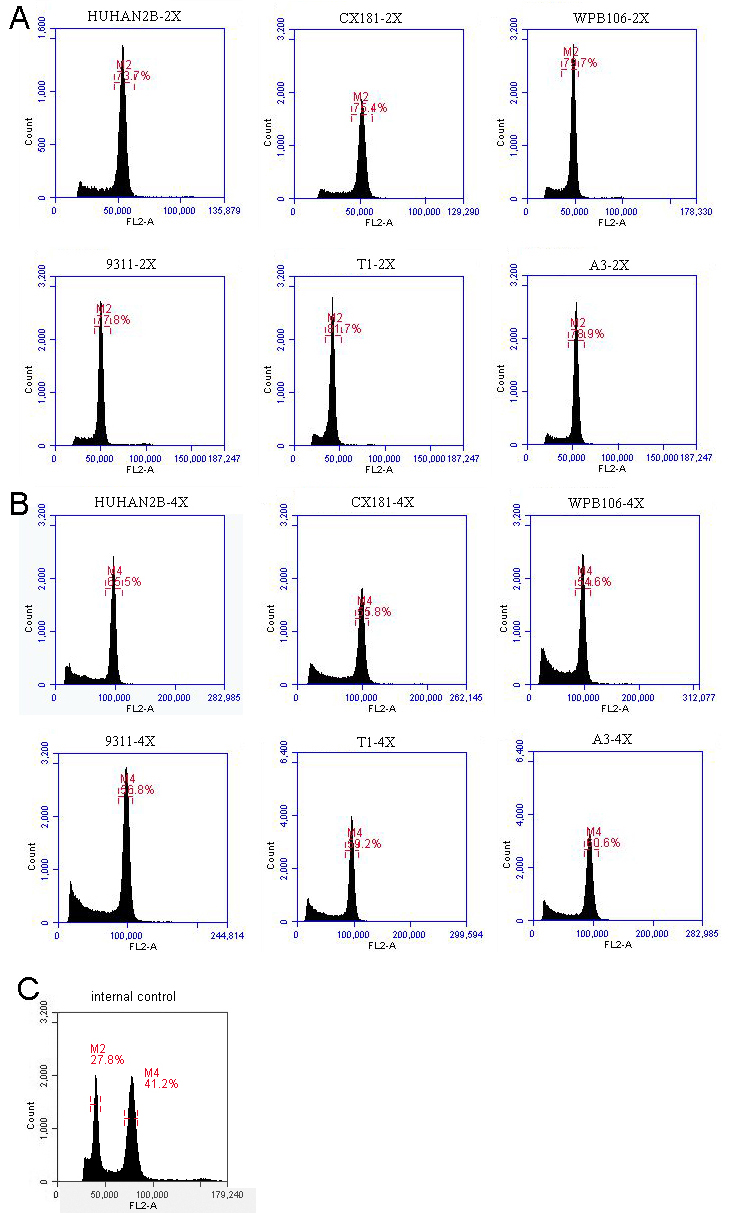

Supplement: Supplementary file 2 — Supplementary Material 2 [file 12284_2024_716_MOESM2_ESM.jpg]

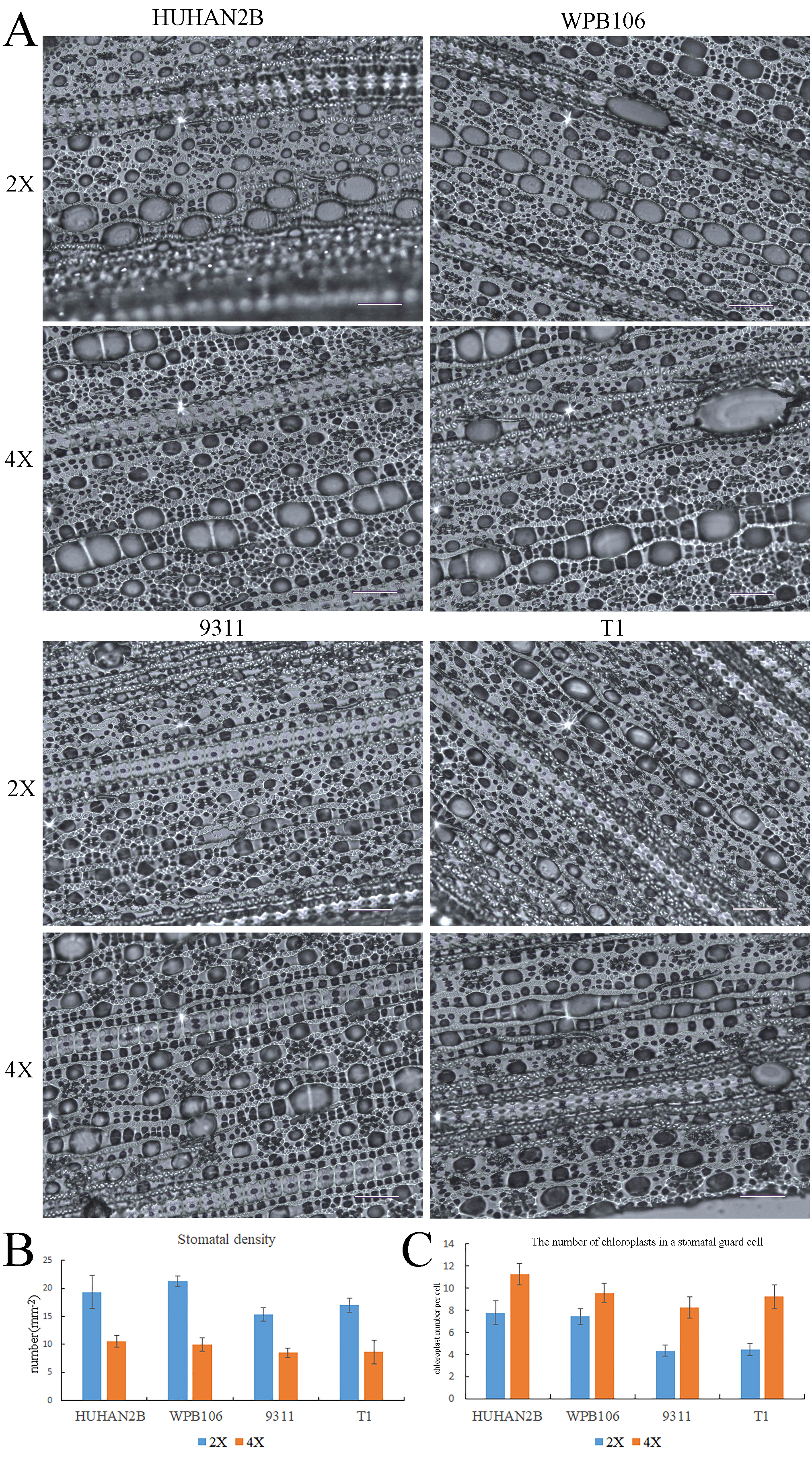

Supplement: Supplementary file 3 — Supplementary Material 3 [file 12284_2024_716_MOESM3_ESM.jpg]

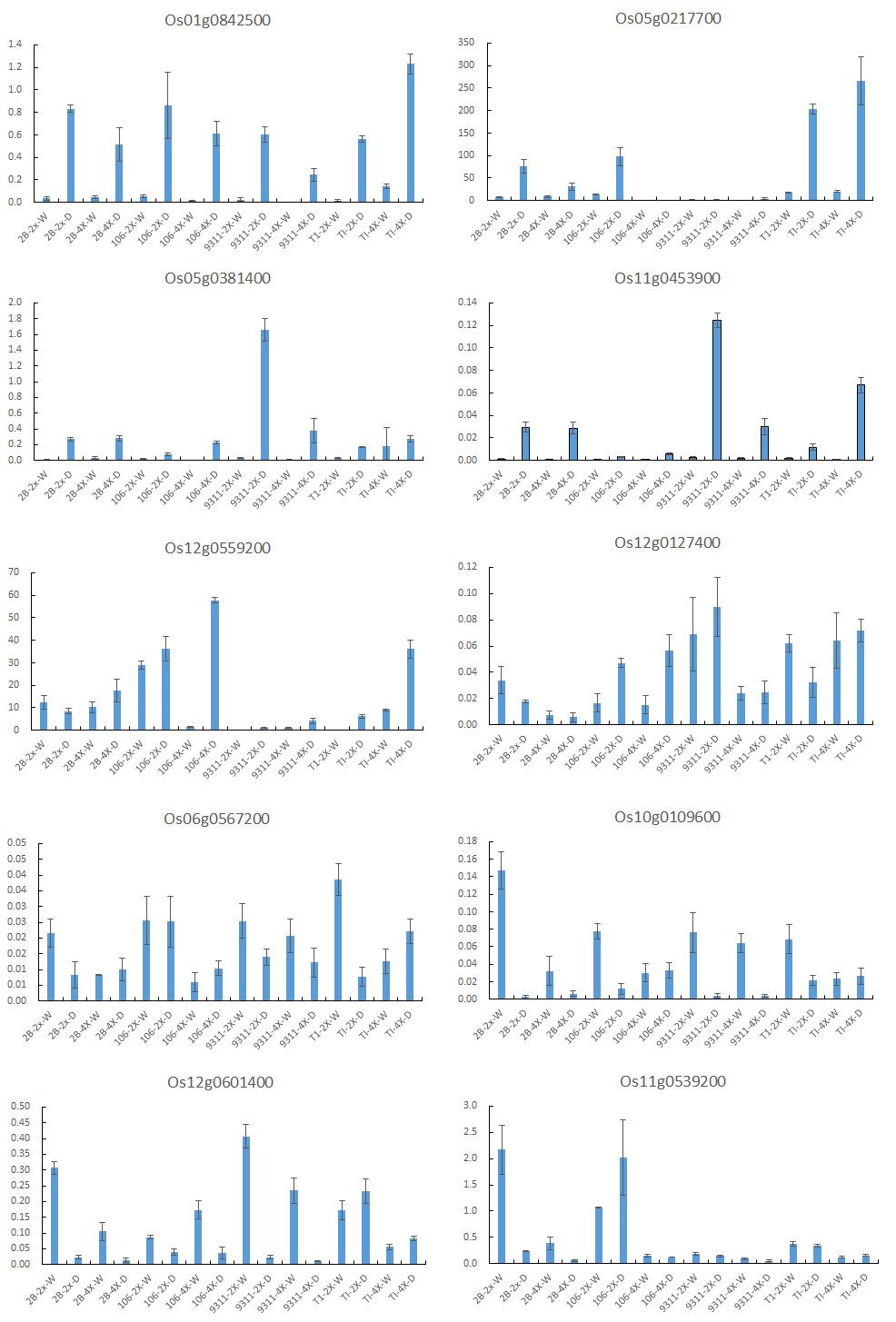

Supplement: Supplementary file 4 — Supplementary Material 4 [file 12284_2024_716_MOESM4_ESM.jpg]

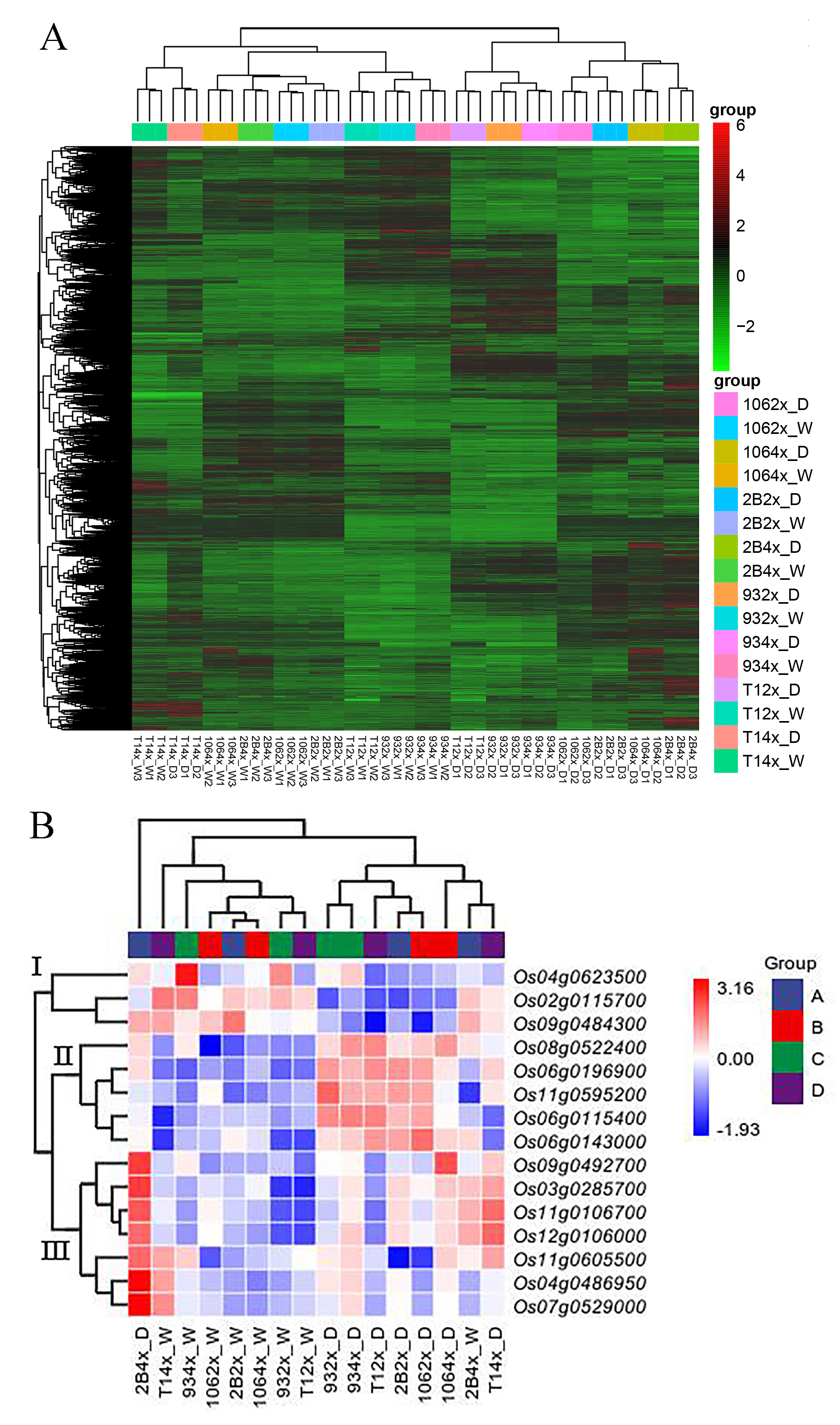

Supplement: Supplementary file 5 — Supplementary Material 5 [file 12284_2024_716_MOESM5_ESM.jpg]

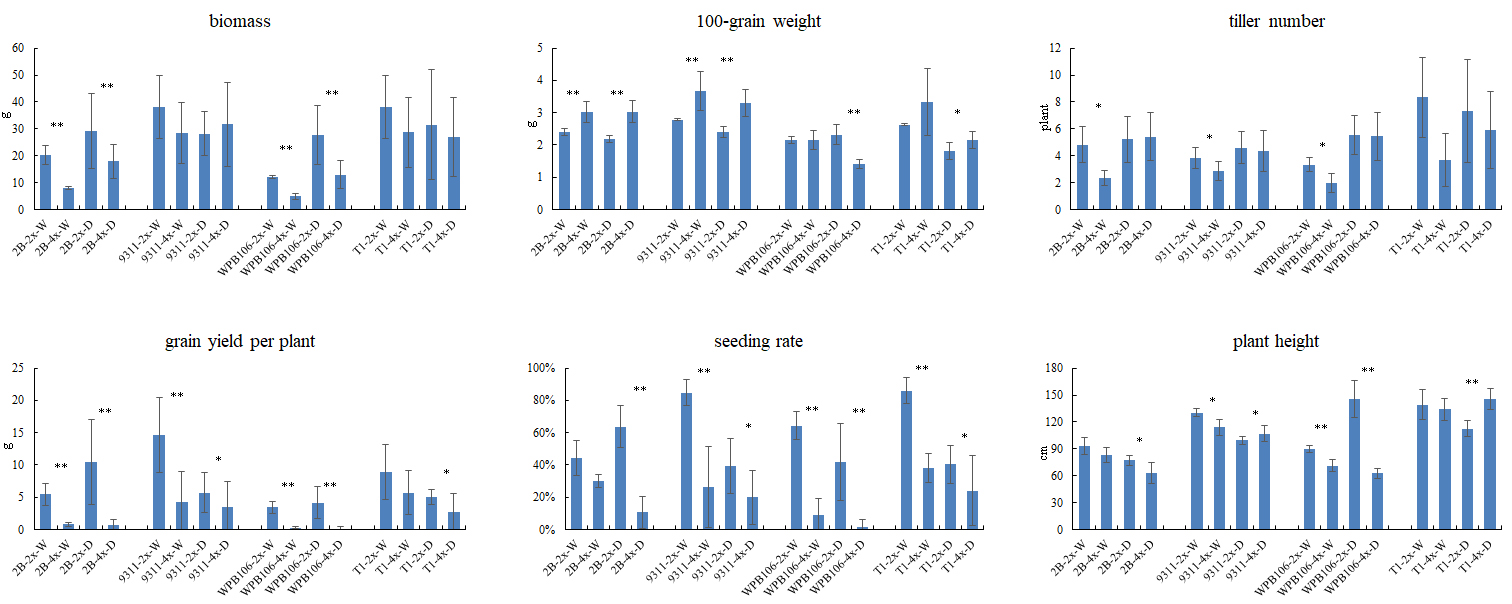

Supplement: Supplementary file 9 — Supplementary Material 9 [file 12284_2024_716_MOESM9_ESM.jpg]
