# Supplementary material for: Autotetraploidy of rice does not potentiate the tolerance to drought stress in the seedling stage
Source: Rice (N Y). 2024 Jun 18;17:40. doi: 10.1186/s12284-024-00716-w (PMC11189374; doi:10.1186/s12284-024-00716-w)
Supplement: Supplementary file 10 — Supplementary Material 10 [file 12284_2024_716_MOESM10_ESM.docx]

Supplementary Material 1：The primers for RT-qPCR.

Supplementary Material 2: Flow cytometry analysis of diploid and tetraploid rice. (A) Diploid plants, (B) tetraploid plants, (C) internal control with diploid and tetraploid cells.

Supplementary Material 3: The stomatal files of diploids and tetraploids. A, the stomatal images. The white arrowheads indicated stomatal files. Bars, 50 μm. 2X, diploid; 4X, tetraploid. B, stomatal density per mm^2^; C, The number of chloroplasts in a stomatal guard cell.

Supplementary Material 4: Relative expression level of DEGs checked by RT-qPCR. 106 indicated WBP106, 2B indicated HuHan2B, 93 indicated 9311 and T1 indicated T1 variety. W, normal condition; D, osmotic stress. 2X, diploid; 4X, autotetraploid.

Supplementary Material 5: Clustering results and Interactive heatmap of differentially expressed genes. (A) Clustering results of differentially expressed genes in the experiment. (B) Interactive heatmap of gene expression related with POD. 106 indicated WBP106, 2B indicated HuHan2B, 93 indicated 9311 and T1 indicated T1 variety. 2X, diploid; 4X, tetraploid. W, normal condition; D, drought condition.

Supplementary Material 6: FPKM of 863 genes from the MElightgreen module.

Supplementary Material 7: Eleven genes of the weighted network diagram in MElightgreen module.

Supplementary Material 8: The new transcripts of photosynthesis system in autotetraploid varieties. + indicates positive strand on chromosome, and - indicates negative strand. j, potentially fragment with at least one splice junction shared with a reference transcript; u, unknown and intergenic transcript; and x, exonic overlap with a reference sequence on the opposite strand.

Supplementary Material 9: Agronomic traits of different varieties in the field. 2X, diploid; 4X, tetraploid. 2B indicated HuHan2B. W, normal condition; D, drought condition.

Supplementary Material 10: the legend of Supplementary Material 1-9.
